# Development of a Double-Antigen Sandwich ELISA for Oz Virus and a Seroepidemiological Survey in Wild Boars in Miyazaki, Japan

**DOI:** 10.3390/pathogens14121288

**Published:** 2025-12-14

**Authors:** Hirohisa Mekata, Mari Yamamoto, Aya Matsuu, Ken Maeda, Haruhiko Isawa, Kentaro Yoshii, Kazumi Umeki, Tamaki Okabayashi

**Affiliations:** 1 Center for Animal Disease Control, University of Miyazaki, 1-1 Gakuen-Kibanadai-Nishi, Miyazaki City 889-2192, Japan; 2 College of Bioresource Sciences, Nihon University, 1866 Kameino, Fujisawa 252-0880, Japan; 3 Department of Veterinary Science, National Institute of Infectious Diseases, Japan Institute for Health Security, 1-23-1 Toyama, Shinjuku-ku, Tokyo 162-8640, Japan; 4 Department of Medical Entomology, National Institute of Infectious Diseases, Japan Institute for Health Security, 1-23-1 Toyama, Shinjuku-ku, Tokyo 162-8640, Japan; 5 Department of Viral Ecology, National Research Center for the Control and Prevention of Infectious Diseases, Nagasaki University, 1-12-4 Sakamoto, Nagasaki City 852-8523, Japan; 6 Department of Internal Medicine, Faculty of Medicine, University of Miyazaki, 5200 Kihara, Kiyotake 889-1692, Japan

**Keywords:** Oz virus, *Thogotovirus*, double-antigen sandwich ELISA, wild boar, seroepidemiological survey

## Abstract

Oz virus, an emerging tick-borne thogotovirus, has been reported to cause fatal human infection in Japan. However, its ecology and geographic distribution remain largely unknown. In this study, we developed a double-antigen sandwich enzyme-linked immunosorbent assay (DAgS ELISA) for detecting Oz virus antibodies in animals and used it to conduct a seroepidemiological survey of wild boars (*Sus scrofa*) in Miyazaki Prefecture, Japan. Recombinant Oz virus nucleoprotein was expressed in *E. coli* and used as both the capture and detection antigen. Relative to the neutralization test, the DAgS ELISA showed a sensitivity of 72.2%, a specificity of 88.2%, and an overall concordance rate of 79.0%. We used this assay to examine 1045 wild boar serum samples collected between November 2022 and May 2025, finding a seroprevalence of 33.5%. The seroprevalence did not significantly differ by sex, age, or region, but showed significant seasonal variation, peaking in summer (*p* < 0.0001). Oz virus RNA was detected by quantitative RT-PCR in one serum sample (0.09%). Phylogenetic analysis of the partial Oz virus glycoprotein gene showed that this strain shared 98.8% nucleotide identity with the EH8 strain, which was the first Oz virus isolate obtained from ticks in Ehime Prefecture. These findings suggest that wild boars in Miyazaki are frequently exposed to Oz virus and that ticks in the region harbor the virus. However, no human cases have been reported to date. The DAgS ELISA developed in this study provides a practical tool for serological surveillance in animals. Continuous monitoring of animal populations is warranted to clarify the epidemiology of Oz virus in the region and to identify potential reservoir species.

## 1. Introduction

**Figure 1:**
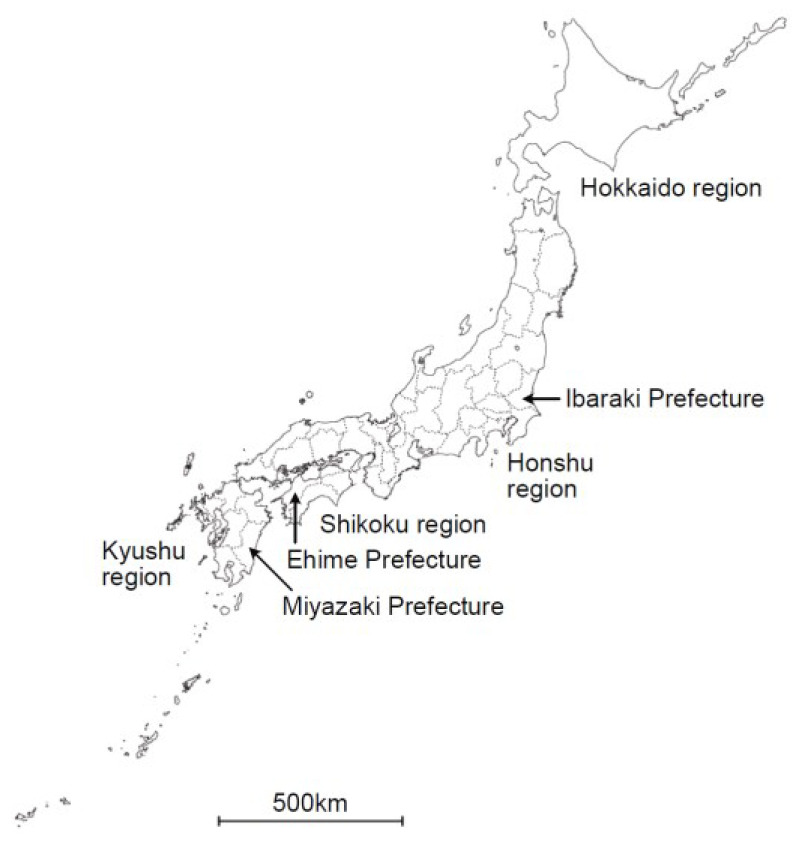
Geographic distribution of Oz virus isolates and sampling site in this study. Map of Japan showing the locations related to Oz virus studies. In this study, wild boar serum samples were collected in Miyazaki Prefecture, located in the Kyushu region. Oz virus has previously been isolated from *Amblyomma testudinarium* ticks in Ehime Prefecture (Shikoku region) and from a human patient in Ibaraki Prefecture (Honshu region). The map also indicates the major geographic regions of Japan (Hokkaido, Honshu, Shikoku, and Kyushu).

Oz virus (*Thogotovirus ozense*) is the causative agent of a novel tick-borne viral disease. It is an enveloped, six-segmented, single-stranded, negative-sense RNA virus classified within the genus *Thogotovirus* of the family *Orthomyxoviridae* [1]. Each genomic segment encodes one of the following proteins: polymerase basic protein 1 (PB1), polymerase basic protein 2 (PB2), polymerase acidic protein (PA), glycoprotein (GP), nucleoprotein (NP), and matrix protein (M). Oz virus was first isolated from ticks collected from vegetation in Ehime Prefecture, Japan, in July 2013 [2] (Figure 1). At the time of this first isolation, no human or animal infections had been reported. However, because Oz virus is genetically closely related to Bourbon virus (*Thogotovirus bourbonense*), which causes fatal tick-borne infections in the United States [3], it was suspected to be pathogenic to humans. As predicted, in June 2023, the first human case was reported in Ibaraki Prefecture, Japan, involving a patient who died of myocarditis caused by Oz virus infection [4]. As of October 2025, this remains the only confirmed human case worldwide. Because of the extremely limited number of reported cases, much about the Oz virus remains unknown. To date, Oz virus has only been isolated from this human patient and from *Amblyomma testudinarium* ticks [2,4]. Thus, *A. testudinarium* is considered a potential natural vector of Oz virus. *A. testudinarium* is a large tick species, and its adults are known to preferentially feed on large mammals such as wild boars and bears, as well as humans [5,6,7]. Indeed, in parts of Honshu (outside northern Japan), wild boars (*Sus scrofa*), sika deer (*Cervus nippon*), and Asian black bears (*Ursus thibetanus*) have been reported to exhibit high prevalence of antibodies against Oz virus, as determined by indirect ELISA or serum neutralization tests [8,9]. Therefore, seroepidemiological surveys of these large wild animals are valuable for investigating the distribution and circulation of Oz virus. When tick-borne infections are suspected, clarifying which pathogens are endemic to the region is crucial for determining the causative agent. In the Kyushu region of Japan, where many tick-borne infectious diseases occur [10,11], epidemiological information on Oz virus in wildlife remains limited.

Serum neutralization tests are considered the gold standard for detecting specific neutralizing antibodies in tick-borne viral infections [12,13]. However, these tests are time-consuming and require handling an infectious virus, making them unsuitable for large-scale screening. Enzyme-linked immunosorbent assays (ELISAs) overcome these limitations. The double-antigen sandwich (DAgS) ELISA is particularly useful for seroprevalence studies across multiple animal species because of its high specificity and cross-species applicability. It has been widely used to investigate the seroprevalence of zoonotic viruses in both humans and animals [14,15,16,17]. The principle of the DAgS ELISA relies on the multivalency of IgM and IgG antibodies. Serum samples are incubated on plates pre-coated with the specific antigen, followed by the addition of an enzyme-labeled identical antigen, which then sandwiches the bound antibodies. Unlike indirect ELISA, this method does not require enzyme-labeled secondary antibodies specific to each animal species. Therefore, the same protocol can be applied to a broad range of animal species, including wildlife species for which species-specific secondary antibodies are difficult to obtain. Furthermore, because the captured antibody is detected by the specific enzyme-labeled antigen, the assay is expected to be more specific than methods that rely on protein A or protein G, which bind nonspecifically to the Fc region of antibodies. Establishing a DAgS ELISA for Oz virus would therefore greatly facilitate investigations into the virus’ prevalence, geographic distribution, and reservoir hosts.

The aim of this study was to develop a DAgS ELISA for Oz virus suitable for seroepidemiological surveys in animals. We then applied this assay, together with real-time reverse transcription PCR (qRT-PCR), to conduct an epidemiological survey of wild boars captured in Miyazaki Prefecture in the Kyushu region of Japan. Although previous serosurveys have mainly focused on Honshu, this study provides the first large-scale dataset from Kyushu, where no human cases have yet been reported. We anticipate that this study will contribute to future epidemiological investigations of Oz virus in animals and a better understanding of its natural transmission cycle.

## 2. Materials and Methods

### 2.1. Animals

A serum from a rabbit immunized with Oz virus was used as a positive control for the neutralization test, the DAgS ELISA, and Western blotting. Eight-week-old female rabbits were purchased from Kitayama Labes Co. (Nagano, Japan). The rabbits were inoculated subcutaneously with 1.76 × 10^7^ copies of the virus. A second inoculation was performed four weeks after the first. One week later, whole blood was collected from the heart under anesthesia, and serum was separated by centrifugation. Additionally, rabbit serum raised against recombinant Oz virus NPs was used as a positive control for Western blotting and the DAgS ELISA. Immunization with recombinant Oz virus NP, as well as subsequent blood collection and serum recovery, was performed by Scrum Inc. (Tokyo, Japan).

Mouse serum containing antibodies raised against thogoto virus (*Thogotovirus thogotoense*) strain Hl-Kamigamo-25 was used to evaluate the antigenic cross-reactivity with Oz virus in the neutralization tests and the DAgS ELISA [18]. Eight-week-old female ICR mice were purchased from Japan SLC (Hamamatsu, Japan). The mice were inoculated intraperitoneally with 2000 plaque-forming unit (PFU) of virus. Four weeks post-inoculation, whole blood was collected from the heart under anesthesia without thoracotomy, and serum was separated by centrifugation.

A total of 1045 serum samples from wild boars (*Sus scrofa*) captured in Miyazaki Prefecture, Japan, between November 2022 and May 2025, were used for the DAgS ELISA and qRT-PCR. Of these, 200 were randomly selected for neutralization tests. Wild boars are designated as harmful wildlife in Japan and are routinely culled by hunters under prefectural control programs. Blood samples were collected by hunters as part of routine surveillance for African swine fever virus and classical swine fever virus. These diagnostic tests were conducted by the Center for Animal Disease Control, University of Miyazaki, under commission from the prefectural authorities, and the residual sera that tested negative for both viruses were made available for this study. The use of residual serum was approved solely for research purposes, specifically for studies on animal infections other than designated notifiable livestock diseases under the Act on Domestic Animal Infectious Diseases Control. Metadata, such as capture location, date, age class (juvenile or adult), and sex, were available for each animal. Miyazaki Prefecture consists of 26 municipalities, conventionally classified into four geographical regions: northern, central, western, and southern. In this study, capture locations were categorized according to these four regions for analysis.

### 2.2. Recombinant Oz Virus NP Production

Double-stranded DNA fragments encoding the full-length sequence of the Oz virus NPs (accession number NC040733), with 15-base In-Fusion cloning sites added at both ends, were synthesized (Integrated DNA Technologies, Coralville, IA, USA). These DNA fragments were inserted into pET6xHN-N or pET6xHN-C vectors (TaKaRa Bio, Kusatsu, Japan) using the In-Fusion cloning method, and the constructs were transformed into competent *E. coli* BL21 (DE3) cells (New England Biolabs, Ipswich, MA, USA) following the manufacturer’s instructions. As a negative control, the pET6xHN-GFPuv vector (TaKaRa Bio), which carries GFPuv downstream of the His-tag sequence, was also transformed into *E. coli*. Sanger sequencing confirmed that there were no amino acid mutations in the His-tagged Oz virus NP insert of the plasmid. Recombinant *E. coli* cells were cultured in LB medium, and protein expression was induced by adding isopropyl β-D-1-thiogalactopyranoside (IPTG; Nacalai Tesque, Kyoto, Japan) to a final concentration of 1 mM. Recombinant *E. coli* cells were lysed in xTractor Buffer with Recombinant DNase I (TaKaRa Bio), and the supernatant was collected by centrifugation. His-tagged recombinant proteins were purified using a HisTalon gravity column (TaKaRa Bio), followed by PD-10 Desalting Columns (GE Healthcare, Chicago, IL, USA), according to the manufacturers’ protocols. The purified recombinant protein was concentrated using an Amicon Ultra Centrifugal Filter, 30 kDa MWCO (Merck, Darmstadt, Germany). The protein concentrations were measured using a Qubit 3.0 Fluorometer (Thermo Fisher Scientific, Waltham, MA, USA). The recombinant Oz virus NPs with His-tags at the N- and C-termini were designated rOzNP-N and rOzNP-C, respectively. The recombinant protein prepared as a negative control was designated as rGFP.

### 2.3. Western Blotting

To evaluate the antigenicity of the recombinant proteins, Western blotting was performed using a fully automated Western blot analyzer (SimpleWestern Abby, Bio-Techne, Minneapolis, MN, USA) as described previously [19]. The recombinant proteins were lysed in 4× Bolt LDS sample buffer (Thermo Fisher Scientific) containing 2% β-mercaptoethanol (Bio-Rad Laboratories, Hercules, CA, USA) and heated at 70 °C for 10 min. Recombinant proteins at final concentrations of 2.5 ng/μL and 5.0 ng/μL were subjected to Western blot analysis. Both a rabbit anti-His-tag polyclonal antibody (Thermo Fisher Scientific) and rabbit serum immunized with Oz virus were used as primary antibodies. The Anti-Rabbit Detection Module (Bio-Techne) was used as the secondary antibody for detection. The expected molecular weights of rOzNP-N, rOzNP-C, and rGFP were 54.8, 55.4, and 27.0 kDa, respectively, as calculated using the ExPASy Compute pI/Mw tool (https://web.expasy.org/compute_pi/, accessed on 28 November 2023).

### 2.4. DAgS ELISA

For the DAgS ELISA, rOzNP-N was labeled with horseradish peroxidase (HRP) using the Peroxidase Labeling Kit-NH2 (Dojindo Molecular Technologies, Kumamoto, Japan), according to the manufacturer’s instructions. The DAgS ELISA was carried out with slight modifications of our previously described DAgS ELISA for SFTS virus [16]. Briefly, 100 μL of 1 μg/mL rOzNP-N solution or phosphate-buffered saline containing bovine serum albumin was added to each well and incubated overnight at 4 °C. After blocking, 100 μL of serum samples (heat-treated at 56 °C for 30 min and diluted 20-fold) were added to both antigen-coated and uncoated wells and incubated at 20–26 °C for 2 h. Following washing, 100 μL of HRP-conjugated rOzNP-N was added to the wells and incubated for 1 h at 20–26 °C. After additional washing, 2,2′-azino-bis-(3-ethylbenzthiazoline-6-sulfonic acid) (ABTS) peroxidase substrate was added and incubated for 30 min at 20–26 °C. The optical density (OD) of each well was measured as the absorbance at 405 nm minus that at 495 nm. The OD value for each sample was calculated by subtracting the OD of the uncoated well from that of the antigen-coated well.

### 2.5. Neutralization Test

An 80% plaque reduction neutralization test (PRNT_80_) was performed to evaluate whether the serum samples contained neutralizing antibodies against Oz virus. The Oz virus EH8 strain was used in this study [2]. Serum samples were diluted 10-fold in PBS and heat-treated at 56 °C for 30 min to inactivate complement activity. Oz virus prepared in DMEM without supplements and adjusted to 400 PFU/mL was mixed with the diluted serum samples in equal volumes. For the virus–PBS control, the virus suspension in DMEM was mixed with an equal volume of PBS without serum and processed in parallel. After incubation of the serum–virus mixture at 37 °C for 1 h, 200 μL of the mixture was applied to Vero cell monolayers in 12-well plates and incubated for 2 h. After washing, the cells were overlaid with maintenance medium containing 1% methylcellulose and incubated at 37 °C in a 5% CO_2_ atmosphere. After 7 days, the cells were fixed with 4% paraformaldehyde for 1 h, washed, and stained with 2% crystal violet. The number of plaques was counted, and the percentage reduction in plaque number relative to the control was calculated. The neutralization test was defined as positive when the plaque reduction ratio exceeded 80%.

### 2.6. RNA Extraction and Detection of Oz Viral RNA

Viral RNA was extracted from 200 μL of wild boar serum using a fully automated nucleic acid extraction system (MagLEAD system, Precision System Science, Chiba, Japan). One-Step PrimeScript III RT-qPCR Mix (TaKaRa Bio) and PrimeTime qPCR assays (Integrated DNA Technologies) with gene-specific primers and probes were used to detect Oz virus and internal control genes. The assay for Oz virus detection was designed in this study based on the M gene of strain EH8 (Accession No. LC320128). Five candidate primer–probe sets were designed using the PrimerQuest Tool (https://sg.idtdna.com/pages/tools/primerquest, accessed on 19 December 2023). The optimal primer pair was selected by melting curve analysis using an intercalating dye, and probes were added to this pair. GAPDH mRNA was used as an internal control, with the primer and probe sequences slightly modified from previous reports for wild boar [16,19]. The primer and probe sequences used in this study were as follows: Oz virus M gene—forward primer: GGAAGCTGGTTCGAGATGATAA; reverse primer: TGTGGGTTAGGGAAATGAAAGA; probe: SUN-TGCGACCTG/ZEN/TATTAGCTCTTCAGAGC-Iowa Black FQ. GAPDH—forward primer: GCTGCCCAGAACATCATCC; reverse primer: GTCAGATCCACRACBRAYAC; probe: ROX-TCACTGGCATGGCCTTCCGT-BHQ2.

### 2.7. Determination of Partial Nucleotide Sequence of Oz Virus GP Gene

For the sample that tested positive for the Oz virus M gene in qRT-PCR, a nested PCR was conducted to amplify a partial fragment of the Oz virus GP gene. The initial RT-PCR was performed using the PrimeScript One Step RT-PCR Kit Ver.2 (TaKaRa Bio). The 10.0 μL reaction mixture contained 5.0 μL of 2 × 1 step Buffer (Dye Plus), 0.4 μL of PrimeScript 1 step Enzyme Mix, forward and reverse primers (0.3 μM each), 2.0 μL of template RNA, and PCR-grade water. The reaction conditions were as follows: reverse transcription at 50 °C for 30 min; initial denaturation at 94 °C for 2 min; and then 50 cycles of 94 °C for 30 s, 55 °C for 30 s, and 72 °C for 60 s. Nested PCR was subsequently performed using 2 μL of the RT-PCR product under the same conditions as RT-PCR, except with a different primer set and omission of the reverse transcription step. The primers were designed with Primer3Plus (https://www.primer3plus.com/index.html, accessed on 22 January 2024) based on the GP gene sequence of Oz virus strain EH8 (Accession No. LC320126). The primer sequences used in the initial and nested PCR were as follows: OzV-GP-1st Fwd, 5′-GCTCATCATACCTGCAATCTTTCC-3′; OzV-GP-1st Rev, 5′-CAGGCTTAGTCTCAGACTTTCAGT-3′; OzV-GP-2nd Fwd, 5′-CAAACAAACTCATCTGGACGTGAA-3′; and OzV-GP-2nd Rev, 5′-ACATACTTGGATGGAGTTGTGACA-3′. Electrophoresis was performed using the E-Gel Power Snap Electrophoresis System with E-Gel EX Double Comb Agarose Gels (2%, Thermo Fisher Scientific). A single band of 383 bp was detected. The amplified fragments were purified with ExoSAP-IT PCR Product Cleanup Reagent (Thermo Fisher Scientific) according to the manufacturer’s instructions. Cycle sequencing was performed using the BigDye Terminator v3.1 Cycle Sequencing Kit (Thermo Fisher Scientific), the OzV-GP-2nd primers, and the purified PCR products. DNA sequencing was conducted using the SeqStudio Genetic Analyzer (Thermo Fisher Scientific). Sequence data were analyzed using ATGC-MAC Ver.7 software (Genetyx, Tokyo, Japan), and primer sequences were removed. The 335 bp partial sequence of strain wild boar/Miyazaki/WB241210-6/2024 has been deposited in GenBank under accession number LC895932.

### 2.8. Phylogenetic Analysis

The partial Oz virus GP sequence obtained in this study, together with available genome sequences retrieved from GenBank, was aligned using ClustalW. Phylogenetic trees were constructed by the neighbor-joining method using MEGA software version 7.0 [20]. Evolutionary distances were calculated using the Kimura two-parameter model. A total of 1000 bootstrap replicates were used to generate trees based on nucleotide sequences of the genome segments.

### 2.9. Statistical Analyses

To compare the ELISA positivity rates among wild boars according to sex, capture region, season, and age class (adult or juvenile), chi-square tests or Fisher’s exact tests were performed depending on sample size. A *p*-value < 0.05 was considered statistically significant. The cutoff value for the ELISA was determined by receiver operating characteristic (ROC) analysis using the DAgS ELISA OD values and PRNT_80_ results (positive or negative). The cutoff was set at the point where the Youden index was maximized [21]. All statistical analyses were performed using GraphPad Prism version 6 (GraphPad Software, San Diego, CA, USA).

## 3. Results

### 3.1. Development of DAgS ELISA

**Figure 2:**
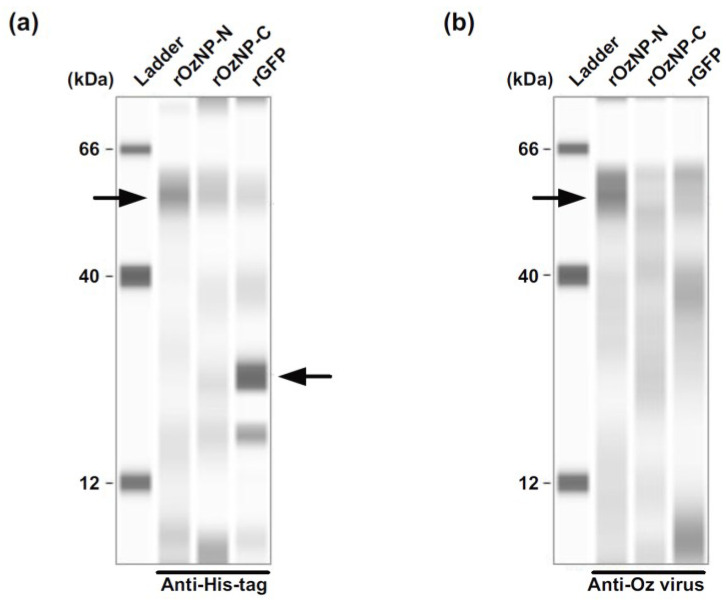
Antigenicity of recombinant His-tagged Oz virus nucleoproteins. Recombinant Oz virus nucleoproteins (NPs) carrying His-tags at the N- and C-termini were designated rOzNP-N and rOzNP-C, respectively. Recombinant His-tagged GFP (rGFP) served as a negative control. (**a**) Purified recombinant proteins were detected using an anti-His-tag polyclonal antibody. (**b**) The antigenicity of the recombinant proteins was evaluated using serum from a rabbit immunized with Oz virus. The expected molecular weights of rOzNP-N, rOzNP-C, and rGFP were 54.8, 55.4, and 27.0 kDa, respectively. Arrows indicate the corresponding protein bands.

**Figure 3:**
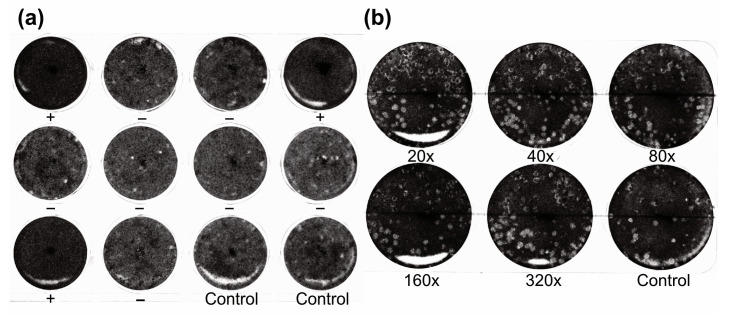
Representative neutralization tests using wild boar serum and mouse antisera against the Hl-Kamigamo-25 strain. (**a**) Representative neutralization test of Oz virus using wild boar serum. Oz virus was mixed with serum at a final dilution of 1:20 and inoculated onto Vero cells in a 12-well plate. Ten wells contained the serum–virus mixture, and two wells served as virus–PBS controls. Plaque formation was examined after 7 days of incubation, and wells showing an >80% reduction in plaque numbers compared with the control were considered neutralization-positive. Neutralization-positive and neutralization-negative wells are indicated by “+” and “−“, respectively, below each well. (**b**) Neutralization test using antisera from mice immunized with the thogoto virus Hl-Kamigamo-25 strain. Serial twofold dilutions (final dilutions of 1:20, 1:40, 1:80, 1:160, and 1:320) were mixed with Oz virus and applied to Vero cells in a 6-well plate, with a virus–PBS control included on the plate. Plaque formation was evaluated after 7 days of incubation. No reduction in plaque numbers relative to the control was observed, indicating no cross-neutralization between Oz virus and the thogoto virus Hl-Kamigamo-25 strain.

**Figure 4:**
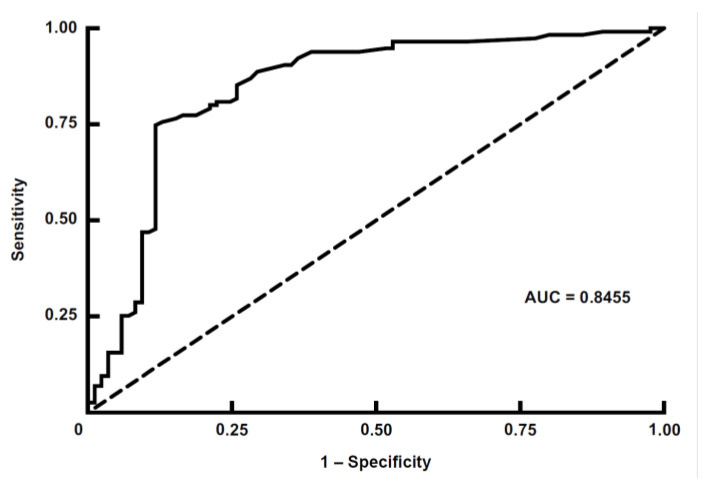
Receiver operating characteristic (ROC) curve analysis of the double-antigen sandwich ELISA for Oz virus. ROC curve showing the diagnostic performance of the double-antigen sandwich (DAgS) ELISA for detecting antibodies against Oz virus. The analysis was conducted using the optical density (OD) values obtained by the DAgS ELISA, with the 80% plaque reduction neutralization test (PRNT_80_) serving as the reference standard. The area under the ROC curve (AUC) was 0.8455, indicating a high discriminative ability of the assay [22]. The optimal cutoff OD value was determined to be 0.0325 based on the Youden index (sensitivity + specificity − 1) [21], yielding a sensitivity of 72.2% and a specificity of 88.2%. The diagonal dashed line represents the line of no discrimination (AUC = 0.5).

To generate recombinant Oz virus NP, expression vectors containing His-tag sequences at both the N- and C-termini of the multiple cloning site were used. The recombinant proteins were expressed in *E. coli*, and their expression was confirmed by Western blotting using an anti-His-tag polyclonal antibody and rabbit serum immunized with Oz virus (Figure 2). When the anti-His-tag antibody was used as the primary antibody, a clear band was observed at approximately 55 kDa for rOzNP-N, whereas the corresponding band for rOzNP-C was relatively faint. The band for rGFP appeared at approximately 27 kDa, as expected (Figure 2a). When rabbit anti-Oz virus serum was used as the primary antibody, a band at approximately 55 kDa was detected for rOzNP-N, but not for rOzNP-C (Figure 2b). Based on these results, rOzNP-N was selected as the antigen for the DAgS ELISA. To optimize the assay, various coating concentrations (5, 4, 3, 2, 1, or 0 µg/mL) of rOzNP-N were tested, and OD values of serially diluted rOzNP-N-immunized rabbit serum were compared. The optimal antigen concentration was determined to be 1 µg/mL, which was used in all subsequent assays. To establish the cutoff value, both the DAgS ELISA and neutralization tests were conducted on 200 wild boar serum samples. Of these, 115 samples showed ≥80% plaque reduction at a 20-fold dilution and were defined as true positives, while 85 samples were defined as true negatives (Figure 3a). An ROC curve was generated based on these results (Figure 4). The area under the ROC curve (AUC) was 0.8455, indicating a high level of discriminative ability [22]. The Youden index was maximized at an OD cutoff of 0.0325 [21]. At this threshold, the sensitivity was 72.2% (83/115), the specificity was 88.2% (75/85), and the overall concordance rate with the neutralization test was 79.0% (158/200) (Table 1).

### 3.2. Verification of DAgS ELISA Sensitivity and Specificity

Because the concordance between the DAgS ELISA and neutralization test was moderate (79.0%), we evaluated potential cross-reactivity with related viruses. The thogoto virus strain Hl-Kamigamo-25 is the only other *Thogotovirus* reported from ticks in Japan [16]. To assess cross-reactivity, mice were immunized with this strain, and the resulting sera were tested using both assays. In the neutralization test against the homologous Hl-Kamigamo-25 strain, 100% plaque reduction was observed at 20-, 40-, and 80-fold dilutions, and 90.4% at a 160-fold dilution, confirming high neutralizing antibody titers. However, when tested against Oz virus, no plaque reduction was observed even at a 20-fold dilution (Figure 3b). Similarly, the DAgS ELISA using rOzNP-N as an antigen yielded an OD of 0.001. These results indicate that neither the DAgS ELISA nor the neutralization test cross-reacted with antibodies against the Hl-Kamigamo-25 strain. Nonetheless, it remains possible that other, as-yet-undiscovered *Thogotovirus* species closely related to Oz virus exist in Japan and that these viruses may cross-react with Oz virus in serological assays.

To evaluate whether the DAgS ELISA might be less sensitive than the neutralization test, we tested serial dilutions of rabbit anti-Oz virus serum using both assays (Table 2). Dilutions from 1:1 to 1:32 were positive in both assays, whereas dilutions from 1:128 to 1:1024 were negative in both. At a 1:64 dilution, the serum was positive by neutralization (PRNT = 94.9%) but negative by ELISA (OD = 0.026), suggesting that the neutralization test is more sensitive. However, since the results were consistent for all other dilutions, the DAgS ELISA was considered sufficiently reliable for the seroepidemiological surveillance of Oz virus infection in wild boars.

### 3.3. Serological Survey of Wild Boars Using the DAgS ELISA

Using the established DAgS ELISA, we analyzed 1045 wild boar serum samples collected in Miyazaki Prefecture, Japan, between November 2022 and May 2025. The overall seroprevalence of Oz virus was 33.5% (350/1045) (Table 3). The regional seroprevalence rates were as follows: northern region, 33.0% (107/324); central, 34.4% (98/286); western, 34.8% (121/348); and southern, 27.5% (22/80). By sex, the seroprevalence rates were 31.2% (177/567) in males and 36.2% (169/467) in females. By age, the seroprevalence rates were 31.8% (28/88) in juveniles and 33.7% (319/946) in adults. Seasonally, the seroprevalence was 28.3% (28/99) in spring (March–May), 46.8% (145/310) in summer (June–August), 32.9% (112/340) in autumn (September–November), and 22.0% (65/296) in winter (December–February) (Table 4). No significant differences were observed by region (*p* = 0.65; chi-square test), sex (*p* = 0.098; Fisher’s exact test), or age (*p* = 0.81; Fisher’s exact test). In contrast, the seroprevalence differed significantly among seasons (*p* < 0.0001; chi-square test). The annual seroprevalence was 36.2% (21/58) in 2022, 31.7% (120/378) in 2023, 36.2% (196/541) in 2024, and 19.1% (13/55) in 2025. The lower rate in 2025 likely reflects the fact that sampling occurred only from January to May, when seroprevalence tended to be lower.

### 3.4. RT-PCR in Wild Boars and Phylogenetic Analysis of Oz Virus

**Figure 5:**
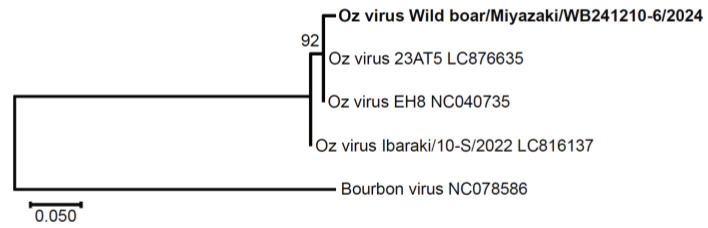
Phylogenetic analysis of Oz virus based on the partial GP gene sequence. A molecular phylogenetic tree was constructed by the neighbor-joining method using partial GP gene sequences of Oz virus strains and Bourbon virus, the closest known relative within the genus *Thogotovirus*. Bootstrap values (1000 replicates) are shown at the major nodes. The scale bar represents 0.05 nucleotide substitutions per site. The strain identified in this study, wild boar/Miyazaki/WB241210-6/2024, is shown in bold. Strains 23AT5 and EH8 were isolated from *Amblyomma testudinarium* ticks collected in Ehime Prefecture, Japan, whereas Ibaraki/10-S/2022 was isolated from a human patient in Ibaraki Prefecture, Japan. GenBank accession numbers are indicated at the end of each reference strain name.

Given the high seroprevalence, we attempted to detect Oz virus RNA in all serum samples using qRT-PCR. One sample (0.09%, *n* = 1045) tested positive, with a quantification cycle (Cq) value of 38.9. To confirm this result, a 383 bp fragment of the partial GP gene was amplified by nested PCR. The obtained sequence, designated wild boar/Miyazaki/WB241210-6/2024, showed 98.8% identity (331/335 nt) with strain EH8 (originally isolated from *A. testudinarium* ticks) and 97.8% identity (328/335 nt) with strain Ibaraki/10-S/2022, which was isolated from the first human case (Figure 5).

## 4. Discussion

In this study, we developed a double-antigen sandwich (DAgS) ELISA for detecting antibodies against Oz virus NP. The sensitivity and specificity of the assay, as determined by comparison with the neutralization test, were 72.2% and 88.2%, respectively (Table 1). Using this ELISA, we investigated the seroprevalence of Oz virus among wild boars captured in Miyazaki Prefecture, Japan, from 2022 to 2025, and found that 33.5% (350/1045) of the animals were seropositive (Table 3). Despite this high seroprevalence, viral RNA was detected in only one sample (0.09%) by qRT-PCR. This suggests that active viremia is rare in wild boars. Phylogenetic analysis of the partial GP gene sequence revealed that the detected strain was more closely related to the Ehime strain than to the Ibaraki strain (Figure 4). Overall, this study established a useful tool for seroepidemiological surveys of Oz virus in wildlife and demonstrated that the virus is widely distributed in Miyazaki, Japan.

In developing the DAgS ELISA, recombinant Oz virus NP was used as the antigen. Previous studies have shown that serum from mice immunized with Oz virus strongly reacts with NP, with weaker reactivity against M protein and GP in Western blotting [23]. Thus, NP represents a rational choice for antibody detection. However, the duration of NP-specific antibody persistence in animals remains unclear. On the other hand, because GP has not been evaluated as an ELISA antigen, we cannot exclude the possibility that Oz virus GP may actually be more suitable for this purpose. One potential advantage of using GP as the ELISA antigen is that it might yield better concordance with neutralization test results. In our Western blot analyses, recombinant NP with an N-terminal His tag was strongly recognized by Oz virus-immunized rabbit serum, whereas the C-terminally tagged NP showed no reactivity (Figure 2b). These findings suggest that C-terminal tagging may alter NP conformation and hinder antibody recognition.

The concordance between DAgS ELISA and neutralization test results was 79.0% (158/200) (Table 1). Notably, 27.8% (32/115) of samples showed negative results for ELISA but positive results for the neutralization test. This discrepancy could be explained by several factors. First, insufficient ELISA sensitivity is likely a major contributing factor, as illustrated by Oz virus-immunized rabbit serum, which was negative according to ELISA at a 1:64 dilution but remained positive according to the neutralization test (Table 2). Second, the heat treatment of sera at 56 °C for 30 min prior to testing may have affected antibody detection. For SARS-CoV-2, heat inactivation has been reported to particularly impair IgM detection, reducing ELISA sensitivity [24,25]. Because the DAgS ELISA format relies more heavily on intact antigen–antibody interactions than the indirect ELISA, it may be even more susceptible to such effects. Although serum heat treatment was implemented for biosafety and consistency with the neutralization test—because wild boar samples may harbor hepatitis E virus [26], Japanese encephalitis virus [27], and other zoonotic pathogens—omitting this step might enhance assay sensitivity in future applications. Third, cross-reactivity with other *Thogotovirus* species cannot be excluded. Based on genomic analyses, *Thogotovirus* species are divided into THOV-like and DHOV-like groups [23]. Oz virus belongs to the DHOV-like group, whereas the thogoto virus strain Hl-Kamigamo-25 is THOV-like. In this study, antiserum against Hl-Kamigamo-25 neither neutralized Oz virus nor reacted in the ELISA, confirming that these viruses are antigenically distinct. Nevertheless, other, as-yet-undiscovered *Thogotovirus* species belonging to the DHOV-like group in Japan could influence serological results. Indeed, antiserum against Bourbon virus has been shown to cross-neutralize Oz virus [23], and several novel tick-borne viruses have recently been identified in Japan [28,29,30,31]. Considering these possible factors, we need to further improve the DAgS ELISA’s sensitivity for more accurate serological evaluation.

Wildlife is thought to play an important role in maintaining the presence of Oz virus in nature. *A. testudinarium*, considered the primary vector of the virus, preferentially feeds on large mammals, especially wild boars [6,32]. Therefore, seroepidemiological surveys of wild boars are valuable for elucidating the distribution and circulation of Oz virus. In the present study, the seroprevalence of the virus among wild boars in Miyazaki was 33.5% (350/1045). Seropositive cases have also been reported in wild boars from Oita (19.2%, 5/26) [9], suggesting that Oz virus is widespread across Kyushu. Large-scale surveys of wild boars in other regions have reported seroprevalence rates of 55.8% (*n* = 344) in Yamaguchi Prefecture (2010–2014), 34.8% (*n* = 89) in Wakayama (2007–2013), and 26.1% (*n* = 243) in Ibaraki (2019–2023) [8,33]. These results indicate that the prevalence observed in Miyazaki is comparable to that in parts of Honshu. Unlike in Ibaraki, where regional differences were significant [33], no such differences were observed in Miyazaki. This may reflect variations between regions where the virus is still spreading and those where transmission has reached equilibrium. Interestingly, we observed significant seasonal variation in seroprevalence (*p* < 0.0001). Given the rapid generational turnover of wild boars [34], population-level seroprevalence likely fluctuates seasonally. Although further studies are needed to clarify why seropositivity increases from spring to summer and declines thereafter, a plausible explanation is as follows: in Japan, wild boars give birth mainly in spring, and maternal antibodies in offspring wane by autumn, reducing overall seroprevalence. Concurrently, the blood-feeding activity of adult *A. testudinarium* increases during summer [35], elevating exposure risk and thus the antibody positivity rate. Continuous, year-round surveillance is therefore warranted to minimize seasonal bias.

Despite the high antibody prevalence, viral RNA was detected in only one wild boar (0.09%), with a high Cq value (38.9). Although qRT-PCR targeted the M gene of Oz virus, a band of the expected size was also obtained by nested PCR targeting the GP gene. Sequencing confirmed that this sequence differed from the positive control strain EH8, indicating that the result was not due to contamination or nonspecific amplification. A recent study in Ibaraki similarly reported 26.1% seropositivity but only 0.1% RNA detection [33]. Comparable patterns are seen in Bourbon virus, where viral RNA is rarely detected in wildlife despite widespread antibody positivity [36]. These findings suggest that, while wild boars are frequently exposed to Oz virus through tick bites, viral replication is likely limited. Wild boars may thus help maintain tick populations rather than serving as true reservoirs. To date, Oz virus has only been detected in unfed nymphs of *A. testudinarium* [2,33]. Because the larvae and nymphs of this tick species feed primarily on small mammals and reptiles, these animals may serve as reservoir hosts [37,38]. Further studies are needed to elucidate the reservoir ecology of Oz virus. The DAgS ELISA developed in this study will be a useful tool for antibody surveillance across a broad range of animal species. To further elucidate the circulation of Oz virus, comprehensive multi-host surveillance, including small mammals and other potential reservoir species, should be incorporated into future monitoring plans.

Phylogenetic analysis revealed that the Miyazaki strain differed slightly from those isolated in Ehime and Ibaraki (Figure 4). Although only four strains were compared, the closer relationship between the Miyazaki and Ehime strains is consistent with their shorter geographic distance (280 km vs. 950 km to Ibaraki). In this study, we also designed nested PCR primers capable of amplifying partial GP sequences even from low-virus-load samples, which will facilitate future comparative analyses among Oz virus strains. Because the GP gene is likely to be under the strongest antibody-mediated selective pressure and therefore prone to accumulate mutations, we considered it suitable for comparative analyses of Oz virus strains and designed primers targeting this region. Similarly, in orthomyxoviruses such as influenza A viruses, the surface glycoproteins hemagglutinin (HA) and neuraminidase (NA)—which correspond functionally to the GP of thogotoviruses—are known to accumulate mutations driven by antibody pressure [39,40].

Finally, only one human case of Oz virus infection has been reported to date, and its pathogenic potential in humans remains largely unknown. Moreover, it is unclear whether animals other than humans develop clinical disease following infection. In the case of SFTS virus, infections causing severe or fatal disease in cats, dogs, and cheetahs were recognized several years after the first human cases [41,42,43]. Therefore, continuous surveillance of Oz virus infection is warranted not only in humans and wildlife but also in companion animals, zoo animals, and livestock.

## 5. Conclusions

In this study, we developed a DAgS ELISA suitable for seroepidemiological surveys of Oz virus in animals. The high seroprevalence (33.5%) among wild boars indicates that Oz virus is endemic in Miyazaki Prefecture. Although no human cases have thus far been reported in the region, some caution is warranted. The rarity of viral RNA detection suggests that wild boars help maintain tick populations but may not serve as major amplification hosts of Oz virus. The natural reservoir of Oz virus therefore remains unidentified, highlighting the need for the continuous epidemiological surveillance of animal populations.

## Figures and Tables

**Table 1:** Correlation between the results of the double-antigen sandwich ELISA and the neutralization test.

	PRNT_80_ ^(b)^ Positive	PRNT_80_ Negative	Total
DAgS ELISA positive ^(a)^	83	10	93
DAgS ELISA negative	32	75	107
Total	115	85	200

Diagnostic performance of DAgS ELISA compared with PRNT_80_: sensitivity: 72.2% (83/115); specificity: 88.2% (75/85); concordance rate: 79.0% (158/200); ^(a)^ DAgS ELISA: double-antigen sandwich enzyme-linked immunosorbent assay; ^(b)^ PRNT_80_: 80% plaque reduction neutralization test.

**Table 2:** Correlation between OD values and neutralization test results for serially diluted Oz virus-immune rabbit serum.

Dilution Ratio of Oz Virus-Immune Rabbit Serum (×)	2	4	8	16	32	64	128	256	512
OD value ^(a)^	1.301	0.883	0.415	0.155	0.059	0.026	0.012	0.010	0.007
Plaque reduction rate ^(b)^ (%)	100	100	100	100	100	94.9	69.2	28.2	0

^(a)^ For the DAgS ELISA, sera were further diluted 20-fold, and 100 μL of each dilution was added per well. Samples were considered positive when the optical density (OD) exceeded the cutoff value of 0.033. ^(b)^ For the neutralization test, sera were further diluted 10-fold and mixed with an equal volume of virus prior to cell infection. Samples were judged positive when the percent reduction in plaque formation was ≥80% compared with the no-serum control.

**Table 3:** Seroprevalence of Oz virus antibodies in wild boars by region and demographic characteristics in Miyazaki Prefecture, Japan (2022–2025).

Region/Category	Positive	Negative	Total	Positive Rate (%)
Northern	107	217	324	33.0
Central	98	188	286	34.3
Western	121	227	348	34.8
Southern	22	58	80	27.5
Unknown	2	5	7	28.6
Male	177	390	567	31.2
Female	169	298	467	36.2
Unknown	4	7	11	36.4
Adult	319	627	946	33.7
Juvenile	28	60	88	31.8
Unknown	3	8	11	27.3
Total	350	695	1045	33.5

**Table 4:** Annual and seasonal seroprevalence of Oz virus antibodies in wild boars in Miyazaki Prefecture, Japan (2022–2025).

Region/Category	Positive	Negative	Total	Positive Rate (%)
2022 (November–December)	21	37	58	36.2
2023 (January–December)	120	258	378	31.7
2024 (January–December)	196	345	541	36.2
2025 (January–May)	13	55	68	19.1
Spring (March–May)	28	71	99	28.3
Summer (June–Aug)	145	165	310	46.8
Autumn (September–November)	112	228	340	32.9
Winter (December–February)	65	231	296	22.0
Total	350	695	1045	33.5

## Data Availability

The original data presented in this study are openly available in FigShare at 10.6084/m9.figshare.30588716.
